# COVID-19 Pandemic: Advances in Diagnosis, Treatment, Organoid Applications and Impacts on Cancer Patient Management

**DOI:** 10.3389/fmed.2021.606755

**Published:** 2021-03-29

**Authors:** Chenyang Ye, Lina Qi, Ji Wang, Shu Zheng

**Affiliations:** ^1^Department of Medical Oncology, The Second Affiliated Hospital of Zhejiang University School of Medicine, Hangzhou, China; ^2^Cancer Institute (Key Laboratory of Cancer Prevention and Intervention, China National Ministry of Education), School of Medicine, The Second Affiliated Hospital, Zhejiang University, Hangzhou, China; ^3^Department of Plastic and Reconstructive Surgery, Zhejiang Provincial People's Hospital, People's Hospital of Hangzhou Medical College, Hangzhou, China; ^4^Department of Surgical Oncology, College of Medicine, Sir Run Run Shaw Hospital, Zhejiang University, Hangzhou, China; ^5^Biomedical Research Center and Key Laboratory of Biotherapy of Zhejiang Province, Hangzhou, China; ^6^Reseach Center for Air Pollution and Health, School of Medicine, Zhejiang University, Hangzhou, China

**Keywords:** COVID-19, SARS-CoV-2, diagnostics, treatment, cancer patient, organoid

## Abstract

Coronavirus disease 2019 (COVID-19) caused by the novel severe acute respiratory syndrome coronavirus 2 (SARS-CoV-2) has spread globally and rapidly developed into a worldwide pandemic. The sudden outburst and rapid dissemination of SARS-CoV-2, with overwhelming public health and economic burdens, highlight an urgent need to develop effective strategies for the diagnosis and treatment of infected patients. In this review, we focus on the current advances in the diagnostics and treatment for SARS-CoV-2 infection. Notably, we also summarize some antineoplastic drugs repurposed for COVID-19 treatment and address the diagnostic and therapeutic challenges for oncologists to manage cancer patients in this COVID-19 era. In addition, we emphasize the importance of organoid technology as a valuable experimental virology platform to better understand the pathogenesis of COVID-19 and assist rapid screening of drugs against COVID-19.

## Introduction

**Figure 1:**
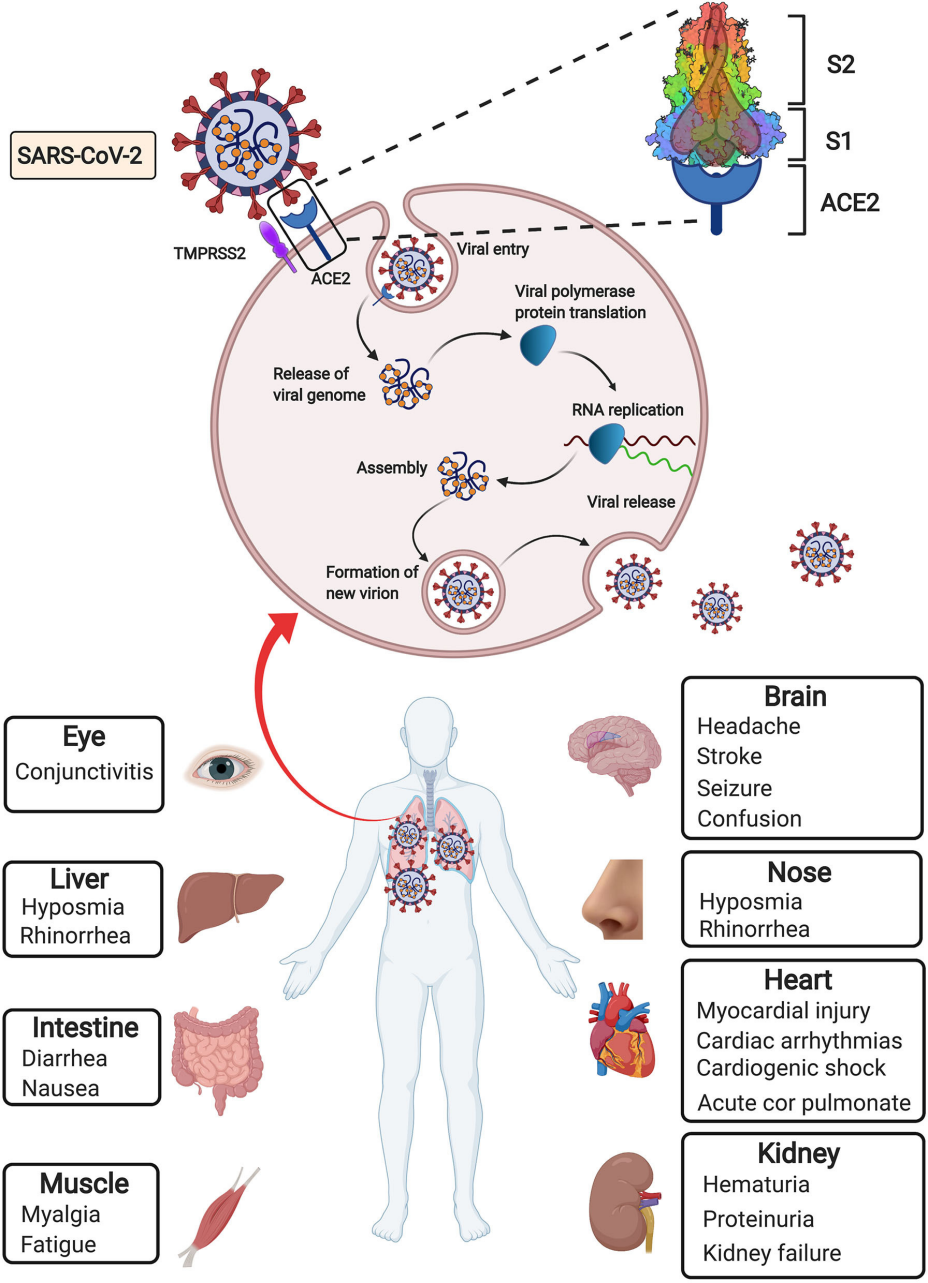
Simplified depiction of SARS-CoV-2 lifecycle and extrapulmonary manifestations of COVID-19. SARS-CoV-2 enters host cells through interaction of its surface spike protein with the ACE2 receptor on the membranes of host cells in the presence of TMPRSS2, which mediates virus–cell membrane fusion and following viral entry. Then viral genomic RNA is released and translated into viral polymerase proteins. Viral RNA is assembled to form mature virions, followed by release of the new virions from the host cells. In addition to the most common pulmonary manifestation of COVID-19, extrapulmonary manifestations derived from many other injured organs have been observed.

In December 2019, coronavirus disease 2019 (COVID-19) caused by the novel severe acute respiratory syndrome coronavirus 2 (SARS-CoV-2) emerged as a new world pandemic (1, 2). As of 9 January 2021, more than 88.9 million cases and 1.91 million deaths have been reported across 188 countries (3), indicating that the SARS-CoV-2 outbreak has become a serious public health emergency of international concern. Coronaviruses, including four genera (Alpha-, Beta-, Gamma-, and Deltacoronavirus), are enveloped, positive-sense, single-stranded RNA viruses that cause infectious diseases in humans and mammals (4). According to phylogenetic analysis of viral genomes, SARS-CoV-2 is a new member of the Beta coronavirus genus, which also includes severe acute respiratory syndrome coronavirus (SARS-CoV). Viral entry into target cells is facilitated by interactions between the spike (S) protein of coronaviruses and the host cell receptor angiotensin-converting enzyme 2 (ACE2) (1, 5–7). Following receptor engagement, the SARS-CoV-2 S protein is primed by cellular serine protease transmembrane protease serine 2 (TMPRSS2) before fusion of the viral and cellular membranes, which is a critical step for the entry and spread of SARS-CoV-2 into host cells (5, 8) (Figure 1).

Since accumulation of SARS-CoV-2 in the respiratory tract is the most serious manifestation, fever and respiratory symptoms, such as cough, shortness of breath, sore throat, etc., are the most common initial symptoms of COVID-19 (9). The impact of COVID-19 goes well beyond the respiratory system to influence the heart and vessels. Several clinical studies showed the correlation between COVID-19 and cardiovascular disease (10, 11). The presence of pre-existing cardiovascular disease is associated with worse prognosis and increased mortality in COVID-19 patients (9, 11, 12). COVID-19 can result in cardiac and vascular complications including acute cardiac injury, myocardial injury, arrhythmia and venous thromboembolism (12, 13). A growing concern over the potential drug-disease interactions in patients with cardiovascular diseases and COVID-19 remains to be solved (14, 15). In addition, SAR-CoV-2 also influences other tissues and organs, such as the brain, eyes, nose, liver, kidneys and intestines (16, 17) (Figure 1). The damage to these organs may manifest specific symptoms, such as seizure, stroke and brain damage, conjunctivitis, diarrhea, hematuria, and oliguria (9).

Given the vast majority of people are still vulnerable to SARS-CoV-2, the development of strategies to diagnose and treat patients with COVID-19 is urgently needed. In this review, we aim to summarize the clinical manifestations of COVID-19 patients, current advances in diagnostic methods and treatment strategies, and organoid applications to fight against COVID-19. Of note, we focus on some repurposing of antineoplastic drugs for COVID-19 and the diagnostic and therapeutic challenges in the management of cancer patients during the current COVID-19 pandemic.

## Diagnostic Strategies for SARS-CoV-2 Infection

Fever and respiratory symptoms are the most common onset symptoms of COVID-19 (9, 18). After screening clinical symptoms and epidemiological history, the highly suspected group required laboratory testing or imaging tests to confirm the COVID-19 diagnosis (19).

After the nucleotide sequence of SARS-CoV-2 was identified from patients' respiratory tract samples by Chinese facilities via deep sequencing analysis (20), a series of detection products based on RT-PCR were obtained. The general process was to sample RNA from the upper respiratory tract, extract RNA, and determine whether it was positive after PCR with a specific primer. There are also serological-based tests. In China, some experts proposed the application of CT imaging to diagnose typical cases in epidemic areas (21), but chest CT screening is not suggested for populations with low infection rates because of its low positive predictive value (22) but may be considered a primary tool for the current COVID-19 detection in epidemic areas (23). In addition to nucleic acid PCR testing and serological testing, there are also tests based on other principles, such as antigen-based testing (24), CRISPR-based methods (25), and physics-based methods (26). One of the main advantages of antigen detection is the fast detection speed. However, antigen detection is very specific to viruses but not as sensitive as molecular PCR tests. SHERLOCK SARS-CoV-2 is short for Specific High-sensitivity Enzymatic Reporter unLOCKing and is based on Cas13a protease and a guide RNA (gRNA) used to recognize a specific new coronavirus genomic sequence (27). No instrument is required, and a simple test similar to a pregnancy test can quickly detect the presence of a new coronavirus RNA sequence using a Sherlock CRISPR SARS-CoV-2 Kit (27, 28). At present, the most widely used detection method is the combination of nasopharyngeal swab nucleic acid PCR and serological IgG/IgM detection (29). Nucleic acid PCR test results are still the gold standard for COVID-19 diagnosis, and serological tests can be used as a supplement (30).

In nucleic acid detection, the sampling site is also critical. The virus can be detected in respiratory, stool, serum (31), urine (32), and sperm samples (33). Saliva or nasopharyngeal swabs are the most convenient to obtain. Doctors use bronchoscopy to sample the lower respiratory tract (34). However, this procedure increases the patient's pain and reduces the efficiency of the test. The kits developed later were mostly nasopharyngeal swabs. At present, there are studies that show that the accuracy of oropharyngeal swab sampling detection may be higher than that of nasopharynx sampling, which further reduces the difficulty of sampling and the patient's pain (35).

As the pandemic began, the requirements for detection time and accuracy were greatly improved. As of 11 May 2020, the FDA had issued 67 individual emergency use authorizations (EUAs) for test kit manufacturers and laboratories for three types of testing (PCR-based testing, serologic testing and antigen testing) (36). The testing time for ID NOW COVID-19 provided by Abbott Laboratories is the shortest at present. Here, we list several typical FDA-approved testing kits and new testing methods in the laboratory stage (Table 1).

**Table 1:** Diagnostic methods for COVID-19.

	**FDA approved**	**Institution**	**Specimen**	**Testing time**	**Notes**
**Virus RNA test**					
TaqPath SARS-CoV-2 Assay	YES	Rutgers Clinical Genomics Laboratory (USA)	Oropharyngeal, nasopharyngeal, anterior nasal, mid-turbinate nasal swab, saliva	n. r	RT-PCR, can detect saliva specimen
TaqPath^™^COVID-19 Combo kit	YES	Thermo Fisher Scientific (USA)	Nasopharynx swab	4 h	RT-PCR
Pixel	YES	Labcorp (USA)	Nasopharynx swab	n. r	RT-PCR, the only home collection kit
Cobas^®^ SARS-CoV-2	YES	Roche (USA)	Nasopharynx swab	3.5 h	RT-PCR
Xpert Xpress SARS-CoV-2	YES	Cepheid (USA)	Nasopharynx swab, nasal wash or aspiratory specimen	45 min	RT-PCR, can run up to 2,000 samples per day
ID NOW COVID-19	YES	Abbott Laboratories (USA)	Nasopharynx swab throat swabs	13 min	ID NOW Instrument based
Bio-Rad SARS-CoV-2 ddPCR Test	YES	Bio-Rad Laboratories (USA)	Nasopharynx swab	5.5 h	RT-ddPCR
BioFire Respiratory Panel 2.1 (RP2.1)	YES	BioFire Diagnostics (USA)	Nasopharynx swab	45 min	Nested multiplex PCR, a multiplexed nucleic acid test
iLACO (isothermal LAMP based method for COVID-19)	NO	Shenyang University (China)	n. r	20 min	RT-LAMP
Sherlock CRISPR SARS-CoV-2 Kit	YES	Sherlock BioSciences, Inc. (USA)	Upper respiratory specimens	<1 h	RT-LAMP+ CRISPR-Cas13 based
CRISPR-based DETECTR assay	NO	Mammoth Biosciences (USA)	Respiratory swab	<40 min	CRISPR-Cas12-based, PPV: 95%, NPV: 100%
Dual-Functional Plasmonic Photothermal Biosensors	NO	Institute of Environmental Engineering (Switzerland)	Respiratory swab	≈17 min	Plasmonic photothermal biosensor based
**Serological test**					
Serology Test qSARS-CoV-2 IgG/IgM Rapid Test	YES	Cellex (Japan)	Serum and plasma	15–20 min	IgG/IgM The first serological test authorized under EUA.
Platelia SARS-CoV-2 Total Ab assay	YES	Bio-Rad Laboratories (USA)	Serum and plasma	n. r	IgM/IgA/IgG specificity> 99%, sensitivity 98%
SARS-CoV-2 IgG assay	YES	Abbott Laboratories (USA)	Serum and plasma	29 min	IgG
Elecsys^®^Anti-SARS-CoV-2	YES	Roche (USA)	Serum and plasma	18 min	IgG Specificity> 99.8%, sensitivity 100%
**Antigen**					
Sofia 2 SARS Antigen FIA	YES	Quidel Corporation (USA)	Nasopharynx swab	<15 min	Test nucleocapsid protein antigen

*n. r, not reported; RT-LAMP, reverse transcriptional loop-mediated isothermal amplification; PPV, Positive predictive value; NPV, Negative predictive value*.

## Therapeutic Strategies Against COVID-19

Given the time-consuming process to develop new drugs starting from scratch, several FDA-approved drugs indicated for other diseases have been repurposed to treat COVID-19 because of their antiviral properties. Notably, some antineoplastic medications have also shown capacities for severe COVID-19 by mitigating hyperactive immune responses and are now being investigated in ongoing clinical trials (Table 2). Here, we summarize the ongoing therapeutic choices, including antiviral drugs, convalescent plasma therapy, and repurposing antineoplastic medications, that are promising to help us fight against COVID-19.

**Table 2:** FDA-approved antineoplastic drugs repurposed for COVID-19 treatment.

**Antineoplastic drugs**	**Mechanism of action**	**FDA approved cancer-specific indications**	**COVID-19 clinical trial identifier**
Tocilizumab	Binds soluble and membrane bound IL-6 receptors, preventing IL-6 mediated pro-inflammatory effect	Cytokine release syndrome	NCT04361552, NCT04331795
Siltuximab	Prevents the binding of IL-6 to both soluble and membrane- bound IL-6 receptors	Multicentric Castleman's disease	NCT04329650, NCT04330638
Imatinib	Multiple tyrosine kinase inhibitor	CML; DFSPs; GIST; ALL; MDS	NCT04357613, NCT04346147
Thalidomide	Immunomodulatory and antiangiogenic effect, suppression of tumor necrosis factor-α	Multiple myeloma	NCT04273529, NCT04273581
Bevacizumab	Monoclonal antibody inhibits the binding of VEGF to its cell surface receptors	Colorectal cancer; Non-squamous non-small cell lung cancer; Glioblastoma; cervical cancer; Renal cell carcinoma	NCT04305106, NCT04275414

*CML, Chronic myelogenous leukemia; DFSPs, Dermatofibrosarcoma protuberans; GIST, Gastrointestinal stromal tumor; ALL, Acute lymphoblastic leukemia; MDS, Myelodysplastic syndrome; VEGF, Vascular endothelial growth factor*.

## Remdesivir

Remdesivir (GS-5734) is a nucleotide analog prodrug that blocks viral replication by inhibiting viral RNA polymerase (37). The therapeutic effectiveness of remdesivir was first evaluated in both cell-based assays and a rhesus monkey model against Ebola virus, in which remdesivir exhibits potent suppression of viral replication and protection from lethal disease (38). However, the efficacy of remdesivir treatment failed to be proven in a randomized controlled human clinical trial in response to a recent Ebola outbreak in the Democratic Republic of Congo (39). Interestingly, a recent *in vitro* study indicated that remdesivir has antiviral activity against SARS-CoV-2 (40). In the case report of the first patient with confirmed COVID-19 in the United States, the patient was intravenously administered remdesivir on hospital day 7 based on the patient's worsening clinical status, including persistent fevers and severe pneumonia. On the 8th day, the patient's clinical condition improved without any adverse events related to remdesivir treatment (41). In a small cohort study of patients with severe COVID-19 who underwent compassionate-use remdesivir treatment, improved clinical outcomes were observed in 36 of 53 patients (68%). However, one clinical trial (ClinicalTrials.gov: NCT04257656) indicated that remdesivir did not exhibit statistically significant clinical benefits compared with those of a placebo (42). But this trial was underpowered due to incomplete full enrollment of eligible patients. The most recent Adaptive Covid-19 Treatment Trial (ACTT-1) was a double-blind, randomized, placebo-controlled trial administrating intravenous remdesivir in 1,062 hospitalized COVID-19 patients (43). The result of this trial showed that remdesivir significantly shortened the time to recovery in COVID-19 patients compared with placebo. However, remdesivir is not routinely recommended in mechanically ventilated COVID-19 patients. Recently, the FDA has approved remdesivir for the treatment of Covid-19 patients requiring hospitalization (44). Because remdesivir alone fails to improve survival rates of COVID-19 patients, several ongoing trials are still awaited to confirm the efficacy and safety of remdesivir combined with modifiers of the immune response for patients with COVID-19 (43, 45).

## Chloroquine and Hydroxychloroquine

Chloroquine (CQ) and hydroxychloroquine (HCQ) (an analog of chloroquine) are two well-known medications used for treating malaria and autoimmune diseases, such as rheumatoid arthritis and lupus (46, 47). Both CQ and HCQ are able to exhibit broad-spectrum antiviral effects by elevating the endosomal/lysosomal pH essential for virus and host cell fusion (47, 48). CQ could also suppress SARS-CoV entry by interfering with the glycosylation of the ACE2 receptor (47, 49, 50). HCQ is typically preferred over CQ due to its better clinical safety during long-term usage, allowance for higher daily dose, and lower potential for drug-drug interaction (51, 52).

Recent *in vitro* studies showed that both CQ and HCQ can effectively control SARS-CoV-2 infection (40, 53). However, in the early stage of the COVID-19 pandemic, there were not enough medical evidence to prove the efficacy of CQ and HCQ treatment for COVID-19, and the results from different small sample studies were controversial (54). Some studies have gained much attention, indicating that HCQ is effective in the treatment of COVID-19 (55, 56). A small open-label non-randomized clinical study from France reported that patients who received 600 mg of HCQ daily had a significant reduction in the viral load. The efficacy of HCQ was reinforced in combination with azithromycin for virus elimination (56). However, the limitations of this study are that comparisons were made between patients at different clinical centers, and six patients (23%) among the 26 HCQ-treated patients were lost to follow-up due to early cessation of treatment, which weakened the conclusion. The same research group later published another study evaluating the effectiveness of HCQ and azithromycin combination therapy in 80 patients. The results showed that 93% of treated patients were negative in nasopharyngeal viral load testing after 8 days (55). However, this study failed to include a control group. Thus, it is unclear whether patients who did not receive HCQ and azithromycin combination therapy would have similar results. It is noteworthy that a prospective study from France failed to obtain any evidence of obvious clinical benefits or strong antiviral effects upon the combination treatment of HCQ and azithromycin for hospitalized patients with severe COVID-19 (57). In their study, 11 patients received the combination therapy of HCQ and azithromycin. However, eight of 10 patients (one patient was not tested due to death) were still positive for SARS-CoV-2 after 6 days. Two patients were transferred to the ICU, and one had to discontinue treatment due to adverse cardiac effects. This study also did not have a control group. Eight of 11 patients had severe comorbidities, including obesity, solid cancer, hematological cancer, and HIV infection, which could be potential confounding effects to influence the results. Similarly, a retrospective study from the U.S. revealed that there was no evidence that therapy with HCQ, either with or without azithromycin, reduced the risk of mechanical ventilation. An association of increased overall death rates was found in patients treated with HCQ alone (58). However, the patients enrolled in this study were all male and over 65 years old (median age), which could introduce bias in this study. In addition, a multicenter, open-label randomized controlled trial including 150 patients in China also concluded that the administration of HCQ did not improve the condition of patients, with a higher negative conversion rate (59). Although the U.S. FDA issued an EUA for the use of HCQ to treat COVID-19 in the United States, the FDA also cautioned against the use of HCQ or CQ for COVID-19 outside of the hospital setting or a clinical trial due to the risk of heart rhythm problems raised by a recent study (60). Therefore, larger high-quality randomized controlled trials are needed to provide a definitive answer regarding the efficacy and safety of this combination. Recently, the controlled, open-label Randomized Evaluation of COVID-19 Therapy (RECOVERY) trial compared the effects between HCQ and usual care in patients hospitalized for COVID-19 (61). Unfortunately, patients who received HCQ treatment did not have better clinical outcomes than those who received usual care. The WHO SOLIDARITY trial also released preliminary results on the efficacy of HCQ in hospitalized patients for COVID-19, and the results were in accordance with the ones from the RECOVERY trial (61). Therefore, HCQ is not an effective treatment for hospitalized patients for COVID-19. The living WHO guideline development panel made a strong recommendation against the use of HCQ for people who are not COVID-19 positive (62). But it remains unclear whether HCQ or CQ could be used in mild-to-moderate COVID-19 cases.

## Lopinavir/Ritonavir

Lopinavir, a human immunodeficiency virus (HIV) type 1 aspartate protease inhibitor, was identified as having an *in vitro* inhibitory effect against SARS-CoV-1 by screening approved drugs for treating severe acute respiratory syndrome (SARS) (63–65). Lopinavir is administered in a fixed-dose combination with ritonavir, a potent CYP3A4 inhibitor, to increase the plasma concentration of lopinavir through the inhibition of cytochrome P450 (64, 66). In an open-label clinical study, treatment with a combination of lopinavir/ritonavir and ribavirin reduced the risk of adverse clinical outcomes (ARDS or death) and viral load among patients with SARS compared with that in a historical control group treated with ribavirin only (64). However, the efficacy of lopinavir/ritonavir was difficult to interpret in that study due to lack of randomization and a contemporary control group and the concomitant use of ribavirin and corticosteroid. Lopinavir was also found to have anti-MERS-coronavirus (CoV) activity both *in vitro* (67) and in a non-human primate animal model (68). Although several clinical case reports indicated that lopinavir/ritonavir (LPV/r)-based combination therapy with ribavirin and interferon alpha led to virological clearance and clinical resolution of infection (69–71), more convincing clinical trial data about the efficacy of this combined therapeutic strategy are needed (71). Therefore, a randomized controlled clinical trial of LPV/r and recombinant interferon-β1b vs. placebo for MERS is currently under way (ClinicalTrials.gov: NCT02845843) (72). Intriguingly, recent research showed that SARS-CoV-2 leveraged species-specific interferon-driven upregulation of angiotensin-converting enzyme 2 (ACE2) to promote infection (the SARS-CoV-2 receptor ACE2 is an interferon-stimulated gene in human airway epithelial cells and is detected in specific cell subsets across tissues). Thus, treatment involving interferon could enhance SARS-CoV-2 infection instead, and caution should be applied in the clinical treatment of patients with COVID-19. For the treatment of severe COVID-19, an open-label, randomized, controlled trial comparing lopinavir/ritonavir (400/100 mg twice daily) (*n* = 99) to standard care (*n* = 100) was performed. The results revealed that lopinavir/ritonavir treatment failed to significantly promote throat viral clearance, facilitate clinical improvement, or reduce mortality in severe COVID-19 patients (66). In addition, one recent study systematically evaluated the clinical characteristics of COVID-19 in patients with liver test abnormalities and found that the use of lopinavir/ritonavir resulted in 4-fold enhanced risk of liver injury (73). The RECOVERY trial is the first large-scale randomized clinical trial to show the effects of lopinavir/ritonavir in patients hospitalized for COVID-19 (74). The result indicated that lopinavir/ritonavir treatment did not reduce duration of hospital stay, risk of progression to invasive mechanical ventilation, or 28-day mortality rate. The interim results of the WHO SOLIDARITY trial also reported that lopinavir–ritonavir did not improve clinical outcomes for COVID-19 patients who require hospitalization (74). Based on the results of recent high quality randomized clinical trials, lopinavir–ritonavir monotherapy is not recommended for patients admitted to hospital with COVID−19.

## APN01

ACE2 has been identified as the key receptor for SARS-CoV both *in vitro* and *in vivo* (75, 76). ACE2 not only acts as the entry receptor of SARS-CoV but also protects against acute lung injury by reducing destructive inflammatory reactions (77). The receptor-binding domain (RBD) of the spike protein of SARS-CoV-2 is very similar to the RBD of SARS-CoV, indicating that both viruses possibly use the common host cell receptor ACE2. Recent studies confirmed that the spike protein of SARS-CoV-2 directly contacts ACE2 to enter cells, and SARS-CoV-2 recognizes human ACE2 even more efficiently than SARS-CoV, suggesting an increased capacity of person-to-person SARS-CoV-2 transmission (6, 78, 79). Treatment with human recombinant soluble ACE2 (hrsACE2) has been proposed to suppress SARS-CoV-2 infections because excessive ACE2 can not only competitively bind with SARS-CoV-2 to block the virus from entering the host cells but also protect the lung from injury by recovering cellular ACE2 activity (80). hrsACE2 could effectively inhibit SARS-CoV-2 replication in Vero cells, engineered human blood vessels and kidney organoids (77). Thus, APN01 (hrsACE2) developed by Apeiron Biologics has undergone a placebo-controlled, double-blinded, phase II clinical trial to evaluate its clinical efficacy and safety in the treatment of COVID-19 patients (ClinicalTrials.gov: NCT04335136).

## Camostat Mesylate

Camostat mesylate (CM), a serine protease inhibitor of TMPRSS2, was developed in Japan primarily for chronic pancreatitis and postoperative reflux esophagitis (81). Since TMPRSS2 is a serine protease that cleaves and activates the spike protein of SARS-CoV-2, which is vital for SARS-CoV-2 entry and viral transmission through interaction with ACE2, CM has become a potential drug candidate for treating COVID-19 (5). Camostat mesylate was validated to inhibit SARS-CoV-2 infection of lung cells, indicating that the host cell entry of SARS-CoV-2 can be effectively inhibited by the clinically proven inhibitor CM. CM is currently undergoing randomized clinical trials (ClinicalTrials.gov: NCT04374019, NCT04355052) that aim to assess whether CM reduces viral entry of SARS-CoV-2 and improves clinical outcomes of patients with COVID-19.

## Baricitinib

Most viruses enter cells through receptor-mediated endocytosis. One of the pivotal regulators of endocytosis is AP2-associated protein kinase 1 (AAK1) (82). Richardson et al. found, using the BenevolentAI machine learning method, a group of AAK1 inhibitors that could suppress clathrin-mediated endocytosis and thereby impair the ability of the virus to infect cells (83). In this study, baricitinib, a Janus kinase (JAK) inhibitor indicated for the treatment of rheumatoid arthritis (RA) (84), was identified with a particularly high affinity for AAK1. Unlike other AAK1 inhibitors, such as the oncology drugs sunitinib and erlotinib, which have serious side effects at the high doses required to inhibit AAK1 effectively, baricitinib can be administered with once-daily oral dosing and trivial side effects (83, 85). In addition, baricitinib has the potential for combination therapy with direct-acting antivirals, such as lopinavir/ritonavir or remdesivir, currently being used and investigated during the COVID-19 pandemic because of its minimal interaction with the relevant cytochrome P450 (CYP) drug-metabolizing enzymes (85). Cantini et al. conducted a pilot study on the safety and clinical efficacy of baricitinib treatment combined with lopinavir-ritonavir in patients with moderate COVID-19 pneumonia (86). However, the limitations of this study, including its open-label, non-randomized feature, lack of properly designed control group, and limited patient number treated with baricitinib, require larger randomized controlled trials to further demonstrate the efficacy of baricitinib treatment.

## Convalescent Plasma Therapy

As a classic passive immunotherapy, convalescent plasma therapy has been used to prevent and treat many infectious diseases since the 1890s (87). Convalescent plasma therapy was successfully applied to the treatment of SARS, H5N1 influenza, 2009 H1N1 pandemic, and MERS, with improved clinical conditions and reduced mortality (88–91). However, in the Ebola virus disease setting, convalescent plasma therapy failed to achieve significant survival improvement (92). Since SARS, MERS, and COVID-19 share similar clinical and virological features (93), convalescent plasma therapy could be a potential treatment alternative for COVID-19 patients (94). One recent laboratory study indicated that sera from several patients can neutralize the COVID-19 virus isolated from the bronchoalveolar lavage fluid of a critically ill patient (1). A systematic review (95) was conducted to assess the clinical efficacy of convalescent plasma therapy for patients with COVID-19. Based on five available clinical studies (87, 96–99), convalescent plasma therapy seems to be promising, with reduced mortality, improved clinical status, and virus clearance. Several randomized clinical trials have been conducted to evaluate the potential benefits of convalescent plasma therapy. Li et al. found convalescent plasma therapy added to standard treatment failed to result in statistically significant improvement in the time to hospital discharge and clinical improvement within 28 days compared with standard treatment in severe or life-threatening COVID-19 patients (100). Another randomized trial in COVID-19 patients with severe pneumonia also observed no significant differences in clinical conditions or overall mortality rates between groups treated with convalescent plasma and placebo (101). But it remains unclear whether convalescent plasma treatment works as a treatment for certain COVID-19 patients incuding mild-to-moderate COVID-19 cases. The RECOVERY trial (Clinical Trials.gov: NCT04381936), the world's largest trial of convalescent plasma is still recruiting COVID-19 patients who do not require invasive mechanical ventilation or extra-corporal membranous oxygenation (ECMO). The completion of RECOVERY trial may provide further evidence about the effectiveness and safety of convalescent plasma treatment.

## Repurposing Anticancer Medications for COVID-19 Treatment

### IL-6 or IL-6 Receptor Inhibitors

Interleukin-6 (IL-6) is upregulated in various solid tumors or hematopoietic malignancies and plays a key role in the initiation and progression of many cancers via the IL-6/JAK/STAT3 pathway (102). Inhibitors targeting IL-6 or the IL-6 receptor have already been used for treating cancers, such as ovarian cancer and metastatic renal cell carcinoma (103, 104). In addition, overwhelmingly elevated IL-6 also plays a central role in cytokine release syndrome (CRS), which can progress quickly to ARDS (105–108). Emerging data indicate that up to 20% of COVID-19 cases develop into ARDS, which is the main cause of mortality in critical patients with COVID-19 (109, 110). Several studies reported that increased serum IL-6 levels were detected in patients with COVID-19 (9, 18) and could serve as an indicator for COVID-19 severity and in-hospital mortality (19, 111, 112). Thus, targeting the IL-6 signaling pathway is a potential therapeutic strategy to control CRS in COVID-19 patients. Tocilizumab, a humanized monoclonal antibody against the IL-6 receptor is currently being used for treating COVID-19 cases with CRS. In one retrospective study of 21 severe and critical COVID-19 patients, tocilizumab effectively improved clinical symptoms and reduced patient mortality without obvious adverse reactions (113). In another study of 100 consecutive patients with COVID-19 pneumonia and ARDS, tocilizumab produced rapid antihyperinflammatory efficacy and remarkable clinical improvement (114). However, the effectiveness of tocilizumab against CRS in the COVID-19 patient setting still needs additional evidence from large randomized, controlled clinical trials. Another humanized anti-human IL-6 receptor monoclonal antibody, sarilumab, and siltuximab, a chimeric antibody targeting IL-6, are currently being evaluated for treating COVID-19 patients with cytokine storm (110). In conclusion, a therapeutic strategy of blocking IL-6 or the IL-6 receptor may be considered a promising choice for the treatment of severe COVID-19 pneumonia and respiratory failure (Table 2).

### Imatinib

Imatinib is an oral anticancer medication used for treating chronic myelogenous leukemia (CML), gastrointestinal stromal tumor (GIST), dermatofibrosarcoma protuberans (DFSPs), and acute lymphoblastic leukemia (ALL) (115). Imatinib plays an inhibitory role in some tyrosine kinase activities, including the oncogenic fusion protein BCR-ABL1 (whose overactivation can result in CML), c-kit (whose mutations are involved in GIST formation), platelet-derived growth factor receptor (PDGFR), and ABL1 kinase (116). In addition, imatinib also displays *in vitro* antiviral capacities against SARS-CoV and MERS-CoV, which are phylogenetically related to SARS-CoV-2 (20, 117). Therefore, imatinib has been postulated to possibly have antiviral function against SARS-CoV-2. In fact, a recent study showed that imatinib binds to the receptor-binding domain (RBD) of SARS-CoV-2 spike protein and inhibits virus replication *in vitro*, indicating imatinib as a potential repurposed drug candidate for COVID-19 treatment (118). In a clinical case report, a patient with COVID-19 pneumonia displayed clinical improvement after receiving imatinib treatment, whereas the clinical condition deteriorated upon hydroxychloroquine (HCQ) and lopinavir/ritonavir (LPV/r) therapy (119). Currently, several ongoing clinical trials are testing the value of imatinib as a promising treatment option for COVID-19 (Table 2). One clinical trial from France (ClinicalTrials.gov: NCT04357613) aims to assess the use of imatinib in aged hospitalized patients with COVID-19. One randomized double-blind trial from the United States (ClinicalTrials.gov: NCT04357613) is evaluating the safety and efficacy of imatinib compared with placebo for the treatment of hospitalized COVID-19 patients. Another randomized, double-blind, placebo controlled, clinical trial from Netherlands (EudraCT2020-001236-10) tries to investigate whether imatinib prevents pulmonary vascular leak in patients with Covid19.

### Thalidomide

Thalidomide was originally given to expectant mothers to alleviate morning sickness between 1958 and 1962 but was later removed from the market due to its serious teratogenicity (120). However, research on the efficacy of thalidomide in other conditions, including cancer, continued, and thalidomide was recently approved by the FDA for treating multiple myeloma (121, 122). In addition, preclinical animal studies showed that thalidomide could alleviate lung injury, with reduced inflammation status and improved survival in mouse models of H1N1 influenza virus infection, indicating the potential therapeutic merit of thalidomide in viral infection (123). Intriguingly, a case report revealed that thalidomide presented an antiviral effect on one patient with COVID-19 (124). The patient with severe COVID-19 received oral thalidomide and low-dose methylprednisolone due to deteriorated clinical manifestations and limited response to other therapies. The patient achieved significant clinical improvement within 1 week of thalidomide treatment (124). However, since this is a single case report, additional clinical studies are needed to confirm the effectiveness of thalidomide and rule out any relevant severe side effects. One clinical trial (ClinicalTrials.gov: NCT04273581) aims to evaluate the efficacy and safety of thalidomide use in combination with low-dose hormones in the treatment of severe COVID-19. Another clinical trial (ClinicalTrials.gov: NCT04273529) is investigating the use of thalidomide in the treatment of patients with moderate COVID-19 pneumonia. Currently, these two clinical trials are still underway evaluating thalidomide therapy in patients with moderate or severe COVID-19 (Table 2).

### Bevacizumab

Vascular endothelial growth factor (VEGF) has been identified as a key molecule in the process of endothelial injury and increases microvascular permeability (125). Higher VEGF levels were observed in COVID-19 patients with ARDS than in healthy people (126). Therefore, VEGF is considered a potential therapeutic target in COVID-19 patients with acute lung injury (ALI) and ARDS. Bevacizumab, a recombinant humanized anti-VEGF monoclonal antibody, is widely used to treat a number of types of solid malignancies, including lung cancer, colon cancer, glioblastoma, and renal-cell carcinoma (127), and is now being evaluated for treating severe or critical patients with COVID-19 pneumonia (Table 2). The result of one clinical trial (ClinicalTrials.gov: NCT04275414) indicated that bevacizumab plus standard care showed remarkable efficacy for treating severe COVID-19 patients (128).

## Current Diagnostic and Therapeutic Challenges in Cancer Patient Care During the COVID-19 Pandemic

Due to the current COVID-19 pandemic, healthcare professionals are facing the overwhelming challenges of rapidly increasing new infection cases, not only to effectively cope with the COVID-19 crisis but also to do so without overlooking the care of patients with other diseases, such as cancer. Cancer patients are more vulnerable to COVID-19 infection and more likely to develop serious events than non-cancer COVID-19 patients due to the immunosuppressive state caused by the cancer itself and anticancer treatments (129–131). Specifically, the rates of severe events in COVID-19-infected patients with hematologic cancer, lung cancer, and metastatic cancers were higher than those in patients without cancer (130). Cancer patients who received surgical or chemotherapy treatments exhibited higher mortality rates and a higher possibility of developing critical symptoms (129, 130). Thus, it is important for oncologists to determine how to properly diagnose and treat cancer patients in this COVID-19 era.

It can be challenging to diagnose whether cancer patients are infected with COVID-19 because some common symptoms of SARS-CoV-2 infection, including fever, dry cough, and shortness of breath, may also be caused by various kinds of cancer. Patients with central-type lung cancer or multiple lung metastases can develop respiratory distress, which often occurs in severe and critical COVID-19 patients (132, 133). Notably, interstitial infiltrate pneumonia displayed by cancer patients who underwent radiotherapy or immune-checkpoint inhibitor treatment could overlap with the symptoms and CT scan characteristics of COVID-19 patients (134–136). Intriguingly, recent studies showed that the levels of some cancer markers, including carcinoembryonic antigen (CEA), carbohydrate antigens (CA) 125 and 153, squamous cell carcinoma antigen (SCCA), and cytokeratin-19 fragment (CYFRA21-1), were elevated in COVID-19 patients and were correlated with the severity of COVID-19 (137, 138).

During the COVID-19 epidemic, medical resources focused on combating COVID-19, fear of nosocomial infection and social distancing led to delay of the daily treatment for cancer patients. For uninfected cancer patients, most nonemergency surgery, intravenous chemotherapy and radiotherapy have been suspended (139). Nonetheless, it is pivotal to maintain medical and surgical treatments for cancer patients (140). Modified management including thorough COVID-19 screening for every cancer patient scheduled for operations, reduced hospital stay, and establishment of virtual connection between patients and their relatives can help reduce cross infection and facilitate safe surgical treatments (140). Many oncologists also use online follow-ups, and switch to oral chemotherapy rather than intravenous administration (141). For elective cancer surgery, COVID-19-free surgical pathways were related with lower pulmonary complication rates, SARS-CoV-2 infection rates, and mortality rates compared with no defined pathway (142). The establishment of COVID-19-free surgical pathways, which provides elective surgery, critical care, and inpatient ward care with no shared areas with COVID-19 patients, is paramount during COVID-19 pandemic (142). Of note, Silvia Fiorelli et al. highlighted the importance that lung cancer patients should continue to receive prompt surgical treatment, and upgraded management strategy is needed for the surgical treatment, patient selection and perioperative management (143). Based on appropriate patient screening and improved precautions, no COVID-19 positive cases were recorded among the medical staff or the hospitalized patients during their hospital stay. Their high-volume thoracic surgery center has successfully maintained safe surgical treatment for lung cancer patients (143). For cancer patients with COVID-19 coinfection, whether to continue antitumor therapy is still controversial. A stable lung cancer patient died rapidly with a history of long exposure to nivolumab immunotherapy (144), but it has also been reported that it is safe to continue targeting in mild cases (145). However, because antitumor therapy will further weaken the immune system and the short-term risk brought by COVID-19 is much higher than the risk of tumors, antitumor therapy for COVID-19-positive cancer patients still needs to be very cautious.

## Applications of Organoid Technology in COVID-19

Organoids are 3D structures that can be generated from adult tissue-specific stem cells, embryonic stem cells, or induced pluripotent stem cells and recapitulate pivotal features of original tissues (146, 147). Organoids provide unique opportunities for modeling and studying human diseases, including congenital and acquired conditions, to establish paradigms for pathogenesis research, high-throughput drug screening, and living organoid biobanks of specific diseases, facilitating personalized treatments (148–150). Cancer patient-derived organoids have been widely used to investigate the mechanism of tumorigenesis and for personalized medicine approaches (151). More importantly, organoids have proven to be ideal models to investigate infectious diseases and the related pathogenic mechanisms (148). Ettayebi et al. successfully modeled human norovirus (HuNoV) infection and propagation using human small intestinal organoids and identified that bile acts as a critical factor for HuNoV replication (152). Similarly, intestinal, lung, gastric, and brain organoids have been applied to model infectious diseases, including Cryptosporidium (153), Middle East respiratory syndrome coronavirus (154), *Helicobacter pylori* (155, 156), influenza virus (157), and Zika virus (158, 159) infections, enabling a better understanding of virus-host interactions, virus pathogenesis and virus transmission.

Currently, limited knowledge of SARS-CoV-2 pathogenesis and transmission is mainly based on clinical features, bioinformatic analysis, and rare autopsy reports (9, 160, 161), in part due to the lack of appropriate *in vitro* cell research models that faithfully resemble host tissues. Therefore, human organoids have been recently adopted by several research groups to investigate the mechanisms of SARS-CoV-2 infection and virus-induced tissue damage (17, 77, 161, 162). Human liver ductal organoids were employed to investigate the infection and liver damage of SARS-CoV-2 and have enabled the identification of liver damage caused directly by viral infection (161). Along the same lines, it has been proven that SARS-CoV-2 can readily infect human intestinal enterocytes, and the host cell membrane-bound serine proteases TMPRSS2 and TMPRSS4 promote the infection process, which indicates that human small intestinal organoids serve as a faithful experimental model for the study of SARS-CoV-2 infection and relevant biology, facilitating future drug testing (17, 162–164). Remarkably, SARS-CoV-2 has been shown to directly infect engineered human blood vessel organoids and kidney organoids, which can be blocked by human recombinant soluble ACE2 (hrsACE2) at early stages of SARS-CoV-2 infection (77).

**Figure 2:**
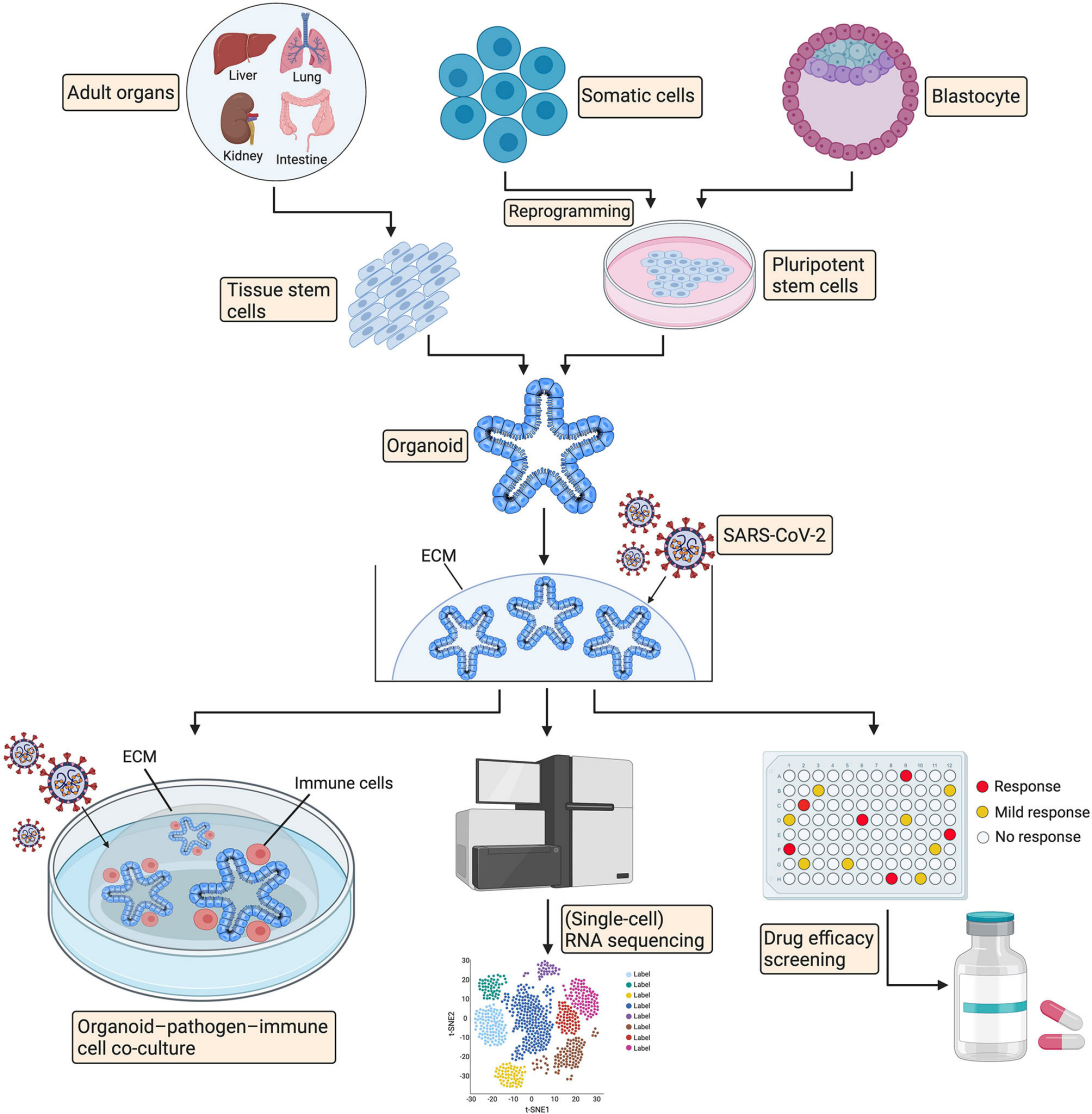
Promising applications of organoid technology in COVID-19. Organoids can be established from adult tissue stem cells, induced pluripotent stem cells, or alternatively, from embryonic stem cells. Organoids can be utilized for pathogenesis investigation via organoid-pathogen-immune cell coculture system and RNA sequencing. Organoids can also facilitate high-throughput drug screening for COVID-19 treatment. ECM, extracellular matrix.

Since SARS-CoV-2 was reported to affect multiple human organs and the underlying mechanisms are still unclear (16), human organoids of the intestinal, lung, kidney, liver, stomach, retinal, brain, and cardiac systems can be leveraged to study pathogenesis in an organ-specific manner (146, 165). In addition, organoid platforms have facilitated personalized drug screening for cancer (146, 166, 167); hence, organoids can also be applied for high-throughput drug screening to discover potential candidates against COVID-19 (Figure 2). Recently, several groups have used organoid-pathogen-immune cell coculture systems to study host–pathogen interactions (168, 169). Organoids were infected with microorganisms (viral or bacterial) before culturing together with immune cells in the triple coculture system (170). In this setting, organoids provide great opportunities to probe the interaction between the epithelium, immune system and SARS-CoV-2 and enable potentially new therapeutic targets for treatment. Further organoid studies for dissecting the pathogenesis of COVID-19 are bound to enable improved understanding and potential drug discoveries (Figure 2).

## COVID-19 Vaccines

Vaccination can efficiently elicit human immunity to prevent infection and disease dissemination, thus helping restrain the SARS-CoV-2 crisis. Multiple methods have been used to generate clinical vaccine candidates for SARS-COV-2, including mRNA vaccines, DNA vaccines, viral vector vaccines, and inactivated virus vaccines (171). Several studies have shown promising immune response inductions and no adverse safety events in Phase III clinical trials (172–175). Currently, over sixty COVID-19 vaccines are being tested in clinical trials, with eleven approved for at least limited use (176). Food and Drug Administration (FDA) have granted three highly effective COVID-19 vaccines for EUAs, including two mRNA vaccines from Pfizer-BioNtech and Moderna, and one adenovirus type 26 (Ad26) vaccine from Johnson & Johnson (177, 178). The two mRNA vaccines require two doses, and second dose should be given within 3 weeks of the first dose for the Pfizer-BioNtech vaccine and within 4 weeks for the Moderna vaccine. Both two mRNA vaccines require ultracold storage, making it harder to distribute. The Ad26 vaccine from Johnson & Johnson is the first single-dose COVID-19 vaccine, and has the advantage of being stable at refrigeration temperature (178). Nonetheless, it still takes time for most people to receive the COVID-19 vaccines. And questions also arise around the safety and effectiveness of COVID-19 vaccines in the setting of cancer patients and elderly population. More researches addressing these unclear issues are needed to identify whether cancer patients and elderly people could benefit from COVID vaccines.

## Limitations of this Review

Several limitations also exist in this review. Firstly, we have cited some preprints in the references, because these papers are still under review or awaiting for publication in official journals. Since these preprints have not been peer reviewed, some interpretations and conclusions from them may need further validation. Secondly, we only discussed the diagnostic and therapeutic challenges in cancer patient care in the COVID-19 era. But some patients with autoimmune diseases or organ transplants are also more vulnerable than healthy people. The diagnostic and therapeutic management of these patients is also noteworthy. Lastly, although there are a great number of important papers, ongoing clinical studies and trials, we can only refer to the most important ones in this review based on our limited knowledge.

## Conclusions and Future Perspectives

How to appropriately manage patients with COVID-19 remains a rapidly evolving preventative and therapeutic challenge. And the efficacy and safety of vaccination in cancer patients or elderly people remain unclear. Therefore, doctors are still urgently seeking existing drugs repurposed for treating COVID-19. Although several therapeutic agents mentioned above in this review are encouraging for treating patients with COVID-19, the clinical trials evaluating definite efficacy and risk of adverse events are still underway. Several guidelines of COVID-19 including IDSA (Infectious Diseases Society of America) guidelines, WHO living guidance, COVID-19 rapid guideline, and CDC (Centers for Disease Control and Prevention) guidelines are important references in terms of diagnosis, treatment, prevention of COVID-19 (62, 179–181). In addition, clinical doctors should continually monitor and adjust management strategies as new literature becomes available. However, caution should be taken when interpreting the available clinical data, since many studies are uncontrolled and have not been peer reviewed.

The COVID-19 outbreak challenges oncologists to properly protect cancer patients, who are assumed to be vulnerable to SARS-CoV-2 infection, without jeopardizing the management of cancer treatment. However, there are still multiple unknowns about how to manage cancer patients who might be exposed to potential infection or may have been infected with SARS-CoV-2. It is important to determine whether COVID-19 would negatively influence active cancer therapies and whether antineoplastic treatments might prevent the cytokine storm caused by SARS-CoV-2. Additionally, data about whether tumor stages and disease status have an impact on COVID-19's interactions with cancer and cancer treatments are lacking. Thus, well-designed, multicentered, prospective cohort studies are required to solve these complex COVID-19 puzzles for cancer patients.

Management of highly contagious and potentially fatal COVID-19 has underscored the urgent need to develop efficient diagnosis methods, specific antiviral therapies or vaccines to fight against SARS-CoV-2. In the current era in which cutting-edge technological methods are available, it is pivotal for us to make collaborative efforts to translate basic and innovative science into the discovery of optimal diagnostic and therapeutic options for clinical applications.

## Author Contributions

CY, LQ, and JW conceived this review and collected the literature. CY and JW drew the schematic diagram. CY and LQ prepared the tables and wrote the manuscript. SZ conducted the study supervision and revised the manuscript. All authors read and approved the final manuscript.

## Conflict of Interest

The authors declare that the research was conducted in the absence of any commercial or financial relationships that could be construed as a potential conflict of interest.

## Abbreviations

COVID-19: Coronavirus disease 2019
SARS-CoV-2: Severe acute respiratory syndrome coronavirus 2
SARS-CoV: Severe acute respiratory syndrome coronavirus
S protein: Spike protein
ACE2: Angiotensin-converting enzyme 2
TMPRSS2: Transmembrane protease serine 2
MERS: Middle East Respiratory Syndrome
ARDS: Acute respiratory distress syndrome
ICU: Intensive care unit
SHERLOCK: Specific high-sensitivity enzymatic reporter unlocking
gRNA: Guide RNA
EUA: Emergency use authorizations
CQ: Chloroquine
HCQ: Hydroxychloroquine
HIV: Immunodeficiency virus
SARS: Severe acute respiratory syndrome
LPV/r: Lopinavir/ritonavir
RBD: Receptor binding domain
hrsACE2: Human recombinant soluble ACE2
CM: Camostat mesylate
AAK1: AP2-associated protein kinase 1
JAK: Janus kinase
IL-6: Interleukin-6
CML: Chronic myelogenous leukemia
GIST: Gastrointestinal stromal tumor
DFSPs: Dermatofibrosarcoma protuberans
ALL: Acute lymphoblastic leukemia
PDGFR: Platelet-derived growth factor receptor
VEGF: Vascular endothelial growth factor
ALI: Acute lung injury
CEA: Carcinoembryonic antigen
CA: Carbohydrate antigens
SCCA: Squamous cell carcinoma antigen
CYFRA21-1: Cytokeratin-19 fragment
HuNoV: Human noroviruses
ECM: extracellular matrix
ACTT-1: Adaptive Covid-19 Treatment Trial
FDA: Food and Drug Administration
ECMO: extra-corporal membranous oxygenation
RECOVERY: randomized evaluation of COVID-19 therapy
Ad26: adenovirus type 26
IDSA: Infectious Diseases Society of America
CDC: Centers for Disease Control and Prevention.
